# Bidirectional relationships between pain and alcohol use among older adults: a scoping review

**DOI:** 10.1093/gerona/glaf258

**Published:** 2025-11-21

**Authors:** Lisa R LaRowe, Heily Chavez Granados, Lisa L Philpotts, Ana-Maria Vranceanu, Christine S Ritchie

**Affiliations:** Mongan Institute Center for Aging and Serious Illness, Division of Palliative Care and Geriatric Medicine, Massachusetts General Hospital, Boston, Massachusetts, United States; Department of Medicine, Harvard Medical School, Boston, Massachusetts, United States; Mongan Institute Center for Aging and Serious Illness, Division of Palliative Care and Geriatric Medicine, Massachusetts General Hospital, Boston, Massachusetts, United States; Treadwell Library, Massachusetts General Hospital, Boston, Massachusetts, United States; Center for Health Outcomes and Interdisciplinary Research, Department of Psychiatry, Massachusetts General Hospital, Boston, Massachusetts, United States; Department of Psychiatry, Harvard Medical School, Boston, Massachusetts, United States; Mongan Institute Center for Aging and Serious Illness, Division of Palliative Care and Geriatric Medicine, Massachusetts General Hospital, Boston, Massachusetts, United States; Department of Medicine, Harvard Medical School, Boston, Massachusetts, United States; (Medical Sciences Section)

**Keywords:** Substance use, Addiction, Comorbidity, Geriatric

## Abstract

**Background:**

Pain and alcohol use are highly prevalent and frequently co-occur among older adults. An established reciprocal model suggests that pain and alcohol use interact in the manner of a positive feedback loop. However, older adults have been underrepresented in this work.

**Methods:**

We conducted a scoping review to answer the following research questions: (a) What is known regarding the effects of alcohol use on pain among older adults? and (b) What is known regarding the effects of pain on alcohol use among older adults?

**Results:**

A total of 15 studies describing interrelationships between pain and alcohol use among older adults were identified and described in this review.

**Conclusions:**

Findings provided convergent evidence that pain can motivate alcohol use in older adults. The effects of alcohol use on longer-term pain outcomes are less clear in this population. Major gaps and directions for future research are described.

## Introduction

Pain and alcohol use are highly prevalent and associated with detrimental health effects among older adults.^1-4^ Chronic pain (pain persisting longer than 3 months) affects 38% of adults aged ≥65 years,^4^ and contributes to suffering, disability, social isolation, poorer aging outcomes, and cognitive decline.^5-7^ Alcohol is the most commonly used substance among older adults.^1-3^ Of particular concern to older adults are hazardous alcohol use (ie, drinking that increases risk for harmful consequences, though specific thresholds vary across studies and contexts^8^) and alcohol use disorder (AUD; ie, a condition characterized by an impaired ability to stop or control alcohol use despite adverse social, occupational, or health consequences). These patterns of drinking impede healthy aging by increasing functional impairment and risk for morbidity and mortality from numerous chronic and serious illnesses (eg, cardiovascular disease, liver cirrhosis, cancer, pancreatitis).^9-11^ Despite this, rates of hazardous drinking and AUD have increased substantially among older adults in recent years.^9^^,^^12^

Moreover, most older adults with pain consume alcohol, and up to 28% of older adults with pain engage in hazardous patterns of alcohol use.^13^ One recent analysis of Health and Retirement Study data revealed that over a quarter of adults aged ≥65 with persistent or recurrent pain reported drinking alcohol at least once per week, with nearly half of these individuals engaging in hazardous patterns of drinking.^14^ Other studies have revealed that rates of hazardous drinking are higher in older adults with (vs without) pain,^15-18^ although the evidence is somewhat inconsistent.^13^ Nonetheless, these findings are concerning, given that pain and hazardous drinking may combine to produce synergistic burdens on individuals, providers, and healthcare systems.^19^^,^^20^

Indeed, an established reciprocal model of pain and substance use posits that pain and alcohol use interact in the manner of a positive feedback loop, resulting in the onset, progression, and maintenance of both chronic pain and hazardous alcohol use over time.^19^ Research in this area is typically divided into 2 directions of empirical inquiry: (1) the effects of alcohol use on pain and (2) the effects of pain on alcohol use. In terms of the effects of alcohol use on pain, although alcohol confers acute analgesic effects,^21^ overtime, alcohol use can lead to increased pain severity, pain-related physical impairment, and the exacerbation of chronic pain.^19^^,^^22^^,^^23^ In terms of the effects of pain on alcohol use, pain can be a potent motivator of alcohol use,^24^^,^^25^ and people often report using alcohol for pain relief.^26^

Although a number of prior reviews have examined relationships between pain and alcohol use,^19-21^^,^^27^^,^^28-31^ none of these papers examined bidirectional relationships between pain and alcohol use *among older adults*, specifically. Older adults warrant specific empirical attention.^32^ High rates of multimorbidity and polypharmacy among older adults^33^^,^^34^ may influence bidirectional pain-alcohol effects. Moreover, age-related physiological changes in pharmacodynamics and pharmacokinetics^35-37^ increase sensitivity to alcohol and lead to higher blood alcohol concentration in response to an equivalent alcohol dose.^38-40^ Consequently, older (vs younger) adults are at greater risk for experiencing adverse alcohol-related effects,^38^^,^^41^^,^^42^ and it is possible that comparatively low levels of alcohol may exacerbate medical conditions, such as chronic pain, among this population.^39^

The objective of this paper is to examine what is known about bidirectional relationships between pain and alcohol use among older adults. We conducted a larger scoping review examining interrelationships between pain and alcohol use in older adults. This paper describes findings from studies in older adults that examined the *effects of alcohol use on pain* (eg, acute analgesic effects of alcohol as a risk factor for developing chronic pain), or the *effects of pain on alcohol use* (eg, pain as a motivator of alcohol use, use of alcohol for pain coping, and pain as a risk factor for developing alcohol-related disorders).

## Methods

We conducted a scoping review following the Joanna Briggs Institute (JBI) methodological guidance for scoping reviews.^43^ Methods are reported in accordance with the PRISMA extension for scoping reviews (PRISMA-ScR^44^). The scoping review was preregistered^45^ and aimed to answer 4 primary research questions: (1) What has been estimated to be the prevalence of co-occurring pain and alcohol use among older adults?; (2) What is known regarding the effects of pain on alcohol use among older adults?; (3) What is known regarding the effects of alcohol use on pain among older adults?; and (4) What interventions have been developed/used to address co-occurring pain and alcohol use among older adults?. Evidence related to the first research question has been reported elsewhere.^13^  *The current paper describes evidence related to the bidirectional relationships between pain and alcohol use in older adults (research questions #2 and #3).*

### Eligibility criteria

Eligibility criteria for the larger scoping review have been described elsewhere.^45^ In brief, we considered human studies that defined their sample as “older”, “geriatric”, “elder”, or “aged” adults. Though this search strategy was intended to prioritize samples of individuals who were aged 65 years and older (eg, the MeSH term “aged” is defined as “a person 65 years of age or older”), we did not exclude studies based on the specific age cutoff used. This decision was made for two primary reasons. First, the definition of “older adult” remains debated in the literature, and cutoffs vary widely across settings and are influenced by social, cultural, and economic factors. Second, given the nascent state of the literature, we elected to take a more inclusive approach since strict age thresholds could exclude potentially relevant studies. For this paper, we included studies that reported the effects of alcohol use on pain (eg, acute analgesic effects of alcohol, alcohol as a risk factor for the development or worsening of chronic pain) or the effects of pain on alcohol use (eg, pain as a motivator of alcohol use, use of alcohol for pain coping, pain as a risk factor for the development or worsening of alcohol use and related disorders). We included studies that utilized a range of measures to assess pain and alcohol use, as there was substantial heterogeneity in how these constructs are assessed across studies. Our goal was to ensure a comprehensive and inclusive review that captures the breadth of the existing literature, rather than limit findings based on measurement variability.

### Types of sources

Primary literature, including analytical observational studies, descriptive observational studies, experimental and quasi-experimental study designs, and qualitative studies was considered for inclusion. We excluded case series and individual case reports to reduce potential bias in findings. We also excluded secondary literature, letters to the editor, commentaries, essays, conference abstracts, conference papers, books, book reviews, and book chapters.

### Search strategy

The search strategy was informed by research questions and developed in consultation with content experts and a medical librarian. The search strategy aimed to identify studies published in English without date restriction using keywords and subject terms. Databases searched included Ovid Medline, Web of Science Core Collection, PsycInfo, Embase, CINAHL, and Cochrane Central Register of Controlled Trials. The search was initially conducted in February 2023 and was updated in April 2024. Details regarding the search strategy can be found in Supplementary Appendix 1.

### Study selection

All identified citations were uploaded into Covidence for screening.^46^ Duplicates were removed. Two independent reviewers screened titles and abstracts using the predetermined eligibility criteria. Articles that were identified as potentially suitable for inclusion by at least one of the reviewers were reviewed in full. Exclusion justification of full texts that did not meet the inclusion criteria were recorded. Disagreements were resolved through discussion and/or with an additional reviewer.

### Data extraction

Two independent reviewers extracted data using a template developed by the authors. Discrepancies were resolved by discussion and consensus between reviewers, with involvement of a third team member to resolve disagreements when necessary. Items extracted included: study information (authorship, publication date, etc.), study design, demographics (age, gender, race/ethnicity), and all key findings relevant to the review questions.

## Results

### Sources of evidence

**Figure 1.**
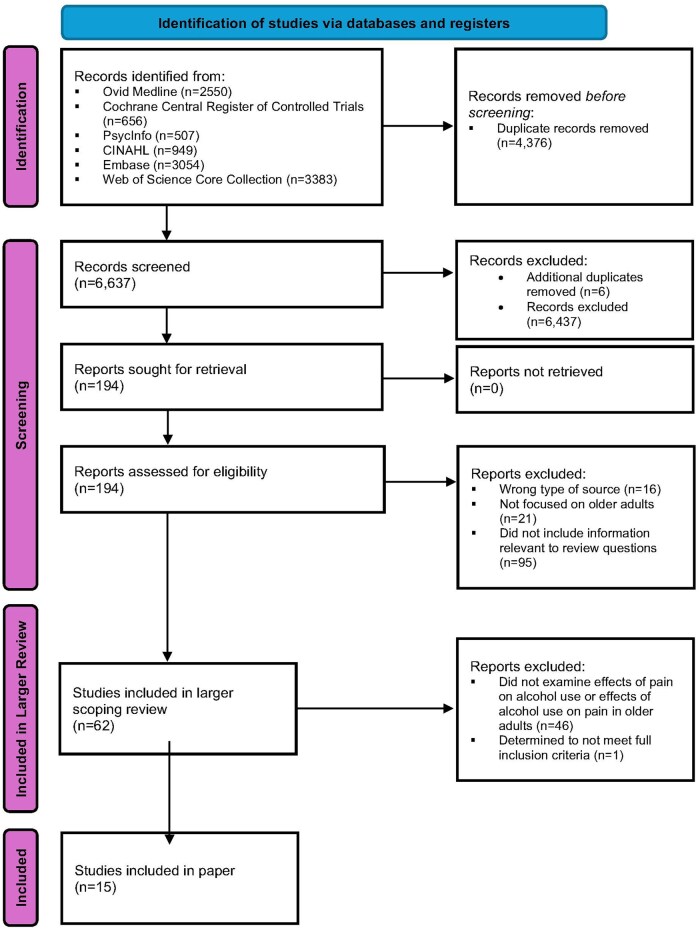
PRISMA flow diagram.

Figure 1 displays the flow diagram for this scoping review. We identified 6637 studies for screening. Of these, 6 additional duplicates were identified and removed. Thus, we reviewed titles and abstracts for 6631 studies. We assessed 194 full texts for eligibility, and 132 did not meet eligibility criteria. Of the 62 studies determined to meet eligibility criteria for the larger scoping review, 16 studies addressed review questions #2 and #3 (ie, “What is known regarding the effects of alcohol use on pain among older adults?” and “What is known regarding the effects of pain on alcohol use among older adults?”). We elected to exclude 1 additional paper from this review^47^ because, although the population was initially described as comprising “aging adults” (thus, we initially felt it met eligibility criteria as described above), those aged 40 and older were included and the “Discussion section” referred to the population generically as “middle-aged and older adults”. Thus, a total of 15 studies were included in the current paper. Of these, 5 studies reported the effects of alcohol use on pain among older adults, 8 studies reported the effects of pain on alcohol use among older adults, and 2 studies reported both.

### Effects of alcohol use on pain in older adults

Table 1 shows results of studies examining the effects of alcohol use on pain in older adults.

**Table 1.** Effects of alcohol use on pain among older adults.

Reference	Country	Study design	Years of data collection	Sample description	Major findings	Alcohol variables	Pain variables
Acute alcohol analgesia							
**Jakobsson 2004**	Sweden	Observational, cross-sectional	2000-2001	294 adults aged ≥75 years who have chronic pain and are in need of help to manage activities of daily living	People living at home reported that alcohol was somewhat helpful for pain management (median = 2 on 1-5 scale), whereas people living in special accommodation reported that it is very helpful (median = 4). People living alone reported that alcohol was somewhat helpful (median = 2) for pain management, whereas people living with someone reported that it is somewhat to mostly helpful (median = 2.5). Alcohol was identified as one of the two most effective methods for pain management among those living in special accommodations.	Participants were asked about alcohol as a pain management method and helpfulness using the Pain Management Inventory (PMI).	Participants were asked about pain that lasts 3 months or longer. Individuals reporting at least “little pain” were asked to complete the Multidimensional Pain Inventory—Swedish version (MPI-S).
**Kuerbis 2019**	US	Observational, longitudinal	2015-2016	55 adults aged ≥50 years who had HIV and chronic pain and screened positive for at-risk substance use	Use of alcohol to cope with pain was not a predictor of relief from pain (*B* = 0.44, *SE* = 0.39, *p* = .27).	Participants were asked how many standard drinks were consumed in the last 24 hours.	Participants were asked if they had experienced pain in the last 24 hours. Participants reporting pain were asked about the worst pain in the last 24 hours, coping with pain and relief from pain were assessed using questions from the Brief Pain Inventory (BPI).
**Risk for developing pain**							
**Caplan 2010**	US	Retrospective cohort study	1998-2006	26 545 veterans aged ≥65 years treated for a vertebral or hip fracture	Alcoholism was not a risk factor for musculoskeletal pain (*p* = .43, HR = 0.94, 95% CI: 0.82-1.09).	Alcoholism status information was obtained from the VA Corporate Franchise Data Center.	Musculoskeletal pain (MSKP) data were obtained from patient records using ICD-9-CM codes compatible with MSKP, including codes for pain without a specific anatomic site (chronic pain), pain syndromes (generalized pain), or pain occurring at multiple sites that are not specified (joint pain, multiple sites).
**Fancourt 2018**	UK	Observational, longitudinal	2004-2005 and 2014-2015	2631 adults aged ≥50 years	Among participants who developed chronic pain, 25.6% reported drinking nearly every day, 40.7% reported drinking 5-6 days per week, 11.4% reported drinking 3-4 days per week, and 22.3% reported drinking 1-2 days per week. In comparison, among those who did not develop chronic pain, 29.3% reported drinking nearly every day, 42.5% reported drinking 5-6 days per week, 11.7% reported drinking 3-4 days per week, and 16.5% reported drinking 1-2 days per week. Lower alcohol consumption was associated with a greater likelihood of reporting chronic pain over the decade (*p* = .001).	Participants were asked about the frequency of alcohol consumption (1-2 days a week, 3-4 days a week, 5-6 days a week, daily).	Participants were asked how often they were troubled by pain and the severity of their pain (mild, moderate, severe). Additionally, participants were asked to specify the location of pain. The study focused on moderate to severe pain reported by participants at any point during the 10-year follow-up.
**Li 2021**	US	Observational, longitudinal	2006-2016	6132 adults aged ≥65 years with moderate to severe chronic pain. Participants were born between 1931 and 1941.	Alcohol consumption (days/week) was not associated with risk for developing moderate to severe chronic pain (RR = 1.00, CI: 0.98-1.03, *p* = .748), but it was identified as a predictor of recovery from high-impact chronic pain (RR = 1.02, 95% CI: 0.99-1.04, *p* = .173). *Significant predictors for recovery from high-impact chronic pain were identified at an alpha level of 0.2, adjusting for sociodemographic variables and duration of previous high-impact chronic pain.	Alcohol consumption was determined from the number of days a participant drank per week.	Participants were considered to have chronic pain if they indicated being troubled by pain often during an interview. They were then asked to rate the severity of pain and pain interference. Individuals reporting moderate or severe pain intensity or pain interfering with their usual activities were classified as having moderate to severe chronic pain. Those reporting no pain or pain without interference in activities were classified as not having moderate to severe chronic pain.
**Marttinen 2021**	Finland	Observational, longitudinal	2002, 2005, 2008, 2012	1954 adults born in 1926-1930, 1936-1940, and 1946-1950	AUDIT-C scores were not associated with odds of remaining in a low pain intensity/low pain interference group (OR = 1.06, *p *= .16).	Weekly alcohol consumption was measured with the AUDIT-C questionnaire.	Frequency and severity of pain were assessed with the bodily pain portion of the SF-36 questionnaire. Pain was categorized into 4 pain intensity pain interference (PIPI) groups: PIPI I for none to mild pain intensity and interference, PIPI II for moderate to severe intensity with none to mild interference, PIPI III for none to mild intensity with moderate to extreme interference, and PIPI IV for moderate to severe intensity with moderate to extreme interference.
**Parreira 2017**	Australia	Observational, longitudinal	2005-2007	1685 men aged ≥70	The odds of reporting persistent pain at 24 months increased with each additional alcoholic drink per week (OR = 1.10, 95% CI: 1.01-1.22; *p = *.03).	Standard number of drinks per week (0-12) was used to measure alcohol consumption.	Participants self-reported the frequency and severity of low back pain in the past 12 months. At the time of enrollment, low back pain was defined as back pain that occurred some, most, or all of the time.

#### Acute analgesic effects of alcohol

Two studies reported the acute pain-relieving effects of alcohol among older adults.^48^^,^^49^ First, in a descriptive cross-sectional study (involving a postal questionnaire and structured personal interviews) of 294 adults aged ≥75 years who had chronic pain and were in need of help to manage activities of daily living, those living at home reported that alcohol was somewhat helpful for pain management.^49^ In contrast, those living in special accommodations (eg, nursing homes, group-dwellings) reported that alcohol was very helpful for pain management, identifying drinking as one of the two most effective methods for pain management. A more recent study collected ecological momentary assessment data from 55 adults aged ≥50 years who had HIV and chronic pain and screened positive for at-risk substance use.^48^ Daily reports of using alcohol to cope with pain did not predict self-reported daily reports of relief from pain (“How much relief did doing the [coping activity/activities] provide?”).

#### Alcohol as a risk factor for pain

Five studies examined alcohol as a risk factor for chronic pain, with mixed findings. A longitudinal study of 1685 older men ≥70 in Australia found that the odds of reporting persistent pain at 2-year follow-up increased by 10% with each additional alcoholic drink per week.^50^ In contrast, a study of 2631 older adults (≥50 years) in the UK revealed that those who developed chronic pain over a 10-year follow-up period were less likely to have consumed alcohol on 5 or more days per week at baseline.^51^ Several other studies found no association between alcohol use and risk for developing pain. First, using clinical and administrative data obtained from clinical encounters during the years of 1998-2006 at the US Department of Veterans Affairs (VA), alcoholism (determined based on administrative codes) was not a significant risk factor for incident musculoskeletal pain among 26 545 US veterans aged ≥65 treated for a vertebral or hip fracture.^52^ Second, a Finnish study of 1954 adults explored variables associated with odds that a participant would remain pain-free over the course of a 10-year follow-up period.^53^ Weekly alcohol consumption (assessed using the Alcohol Use Disorders Identification Test consumption questions) was not associated with pain status. Third, a US study of 6132 older adults (age ≥65) with moderate to severe chronic pain revealed that frequency of alcohol consumption was not associated with risk for developing moderate to severe chronic pain.^54^ However, consuming alcohol on more days per week was associated with greater odds of recovery from high-impact chronic pain within 2 years, after adjusting for sociodemographic variables and the duration of previous high-impact chronic pain.

### Effects of pain on alcohol use in older adults

Table 2 shows results of studies examining the effects of pain on alcohol use.

**Table 2.** Effects of pain on alcohol use among older adults.

Reference	Country	Study design	Years of data collection	Sample description	Major findings	Alcohol variables	Pain variables
Pain as a motivator of drinking							
**Kuerbis 2019**	US	Observational, longitudinal	2015-2016	55 adults aged ≥50 years who had HIV and chronic pain and screened positive for at-risk substance use	The presence of any pain in the last 24 hours was not a significant predictor of same-day substance use (alcohol, marijuana, or other drug use). However, daily rating of worst pain in the last 24 hours was a significant predictor of alcohol use, such that for every one-unit increase in worst pain beyond an individual’s personal mean, alcohol use increased by a quarter drink.	Participants were asked how many standard drinks were consumed in the last 24 hours.	Participants were asked if they had experienced pain in the last 24 hours. Participants reporting pain were asked about the worst pain in the last 24 hours, coping with pain and relief from pain were assessed using questions from the Brief Pain Inventory (BPI).
**Use of alcohol to cope with pain**							
**Begam 2024**	Nepal	Qualitative	2023	20 older adults aged ≥60 in Nepal	Qualitative results showed participants report using alcohol to reduce bodily pain.	Participants were asked if they currently consume alcohol.	Participants were asked about physical pain.
**Brennan 2005**	US	Observational, longitudinal	Not reported	401 adults aged 55-65 who had consumed alcohol at some point in their lives	Among men, 37.9% of problem drinkers report using alcohol to manage pain compared to 15.1% of non-problem drinkers (*p* < .01). Among men with moderate to very severe pain, 56.5% of problem drinkers report using alcohol to manage pain compared to 20.8% of non-problem drinkers (*p* < .01). Among women, 37.9% of problem drinkers report using alcohol to manage pain compared to 12.5% of non-problem drinkers (*p* < .01). Among women with moderate to very severe pain, 58.6% of problem drinkers report using alcohol to manage pain compared to 20.7% of non-problem drinkers (*p* < .01). Among both men and women, more bodily pain and more drinking problems at baseline were independent predictors of using alcohol to manage pain. A total of 49 participants initiated use of alcohol to manage pain over the 3-year follow-up interval and another 49 individuals stopped using alcohol to manage pain over the follow-up interval.	Drinking problems were assessed with the Drinking Problems Index (DPI). At baseline, individuals were classified as current problem drinkers if their DPI responses indicated one or more drinking problems. Participants were asked to indicate how often in the past month they had used alcohol to manage pain (ranging from “never” to “very often”).	Pain was assessed with the global bodily pain item from the Medical Outcomes Study (MOS) survey. Participants were asked to rate the amount of bodily pain experienced in the past month (none to very severe). A question from the MOS survey asked participant to rate the interference of bodily pain with their normal activities.
**Jakobsson 2004**	Sweden	Observational, cross-sectional	2000-2001	294 adults aged ≥75 years who have chronic pain and are in need of help to manage daily activities of daily living	3% of people living at home reported using alcohol for pain management compared to 2% of people living in special accommodation (*p >* .05). 4% of people living alone reported using alcohol to manage pain compared to 2% of people living with someone (*p >* .05).	Participants were asked about alcohol as a pain management method and helpfulness using the Pain Management Inventory (PMI).	Study questionnaire asked about pain that lasts 3 months or longer. Individuals reporting at least “little pain” were asked about their pain using the Multidimensional Pain Inventory—Swedish version (MPI-S).
**Park 2011**	US	Observational, cross-sectional	Not reported	150 adults aged ≥65 years who had chronic pain and were prescribed opioid analgesics	Participants responded to an item stating: “At times, I drink alcohol to help my pain”, using a five-point Likert-type scale ranging from 0 (disagree) to 4 (agree). The modal response was 0, with a mean = 0.13 (*SD* = 0.45) and median = 0.	Participants were asked to respond to an item stating: “At times, I drink alcohol to help my pain”.	Chronic pain was defined as pain that lasts for more than 3 months.
**Riley 2002**	US	Observational, cross-sectional	Not reported	1634 adults aged ≥65 years	When asked what they have done for specific symptoms in the past 12 months, 13% of older adults reported drinking alcohol for jaw joint pain, 24% for face pain, 16% for oral sores, 14% for burning mouth, and 22% for toothache. Male respondents were significantly more likely to report self-treating with alcohol than females for jaw joint pain (33% vs 7%, RR = 5.1, *p* = .01), face pain (54% vs 11%; RR = 5.0, *p < .*01), painful oral sores (44% vs 7%; RR = 6.3, *p* < .01), and toothache pain (56% vs 6%; RR = 9.9, *p* < .01).	Participants were asked how often they drink alcohol (wine, beer, or liquor).	Participants were asked about occurrence of jaw joint pain, face pain, oral sores, burning mouth, and toothache pain during the last 12 months.
**Moos 2010**	US	Observational, longitudinal	Not reported	719 adults aged 55-65 who had consumed alcohol at some point in their lives and had outpatient contact with a healthcare facility in the past 3 years	A total of 20% and 22% of older adults reported using alcohol to manage pain at the 10-year and 20-year follow-ups, respectively. At both follow-up time points, individuals who relied more on alcohol to manage pain drank more frequently and in heavier amounts. Those who relied more on alcohol to reduce pain also reported more warning signs of negative consequences of alcohol consumption and had more drinking problems (*p* < .05). A significant interaction at the 10-year follow-up indicated that reliance on alcohol to reduce pain potentiated the positive association between current alcohol consumption and warning signs of negative alcohol-related consequences. A significant interaction at the 20-year follow-up indicated that reliance on alcohol to reduce pain moderated and potentiated the positive association between alcohol consumption and drinking problems.	Participants were asked to rate how often the individual had used alcohol to reduce pain in the past month. Frequency of alcohol consumption was determined based on how often participants consumed alcoholic drinks per week. Quantity of alcohol consumption was measured by asking participants the largest amount of alcohol consumed on one day in the past month. Individuals with no alcohol consumption in the past year prior to the study were considered abstainers.	Participants were asked about pain in the last 12 months.
**Pain as a risk factor for developing/worsening alcohol use**							
**Bobo 2013**	US	Observational, longitudinal	1998-2008	3105 men aged 50-65 years in 1998 who completed at least 3 interviews from 1998 to 2008	A history of frequent pain significantly increased the likelihood that older men would report decreasing alcohol consumption (vs at-risk drinking) at follow-up (OR = 1.83, 95% CI: 1.17-2.88). A history of frequent pain also significantly increased the likelihood that older men would report decreasing alcohol consumption (vs moderate drinking) at follow-up (OR = 1.63, 95% CI: 1.12-2.37).	Participants that indicated recent drinking were asked about drinking habits in the past 3 months: which days of the week individuals drink on average, how many drinks are consumed on those days, and if more than 4 drinks were consumed on one occasion.	Participants were asked if they were often troubled by pain.
**Brennan 2011**	US	Observational, longitudinal	Not reported	1291 adults aged 55-65 who reported at least occasional alcohol use and had contact with outpatient clinics within the past 3 years	Baseline number of painful medical conditions alone had no effect on the 10-year pattern of decline in drinking problems. However, interpersonal spouse/partner resources moderated the effect of painful medical conditions on 10-year change in drinking frequency, such that, among individuals with more spouse/partner resources, having more painful medical conditions at baseline hastened decline in drinking frequency, whereas they had no effect on drinking frequency of individuals with fewer spouse/partner resources. Age also moderated the relationship between baseline painful medical conditions and 10-year change in frequency of drinking problems, with painful conditions hastening the decline in drinking problem frequency in the slightly older (ie, age 61-65 at baseline) participants but having no effect in the younger (age 55-60) participants.	Frequency of drinking was determined using 3 questions from the Health and Daily Living Form about weekly consumption of wine, beer, or distilled spirits in the last month. This was asked at baseline, 1-year, 4-year, and 10-year follow-ups. Frequency of drinking problems was assessed using the Drinking Problem Index.	Painful medical conditions were assessed using items in the health stressors subscale from the Life Stressors and Resources Inventory (LISRES). Participants were asked if they had experienced painful medical conditions (including back pain, chest pain, headache, stomach pain, and joint pain) in the past year.
**Brennan 2013**	US	Observational, longitudinal	1998, 2000, 2002, 2004	5446 adults aged 55-65	More numerous painful medical conditions had no effect on the 8-year rate of change in participants’ likelihood of consuming alcohol. Effects of baseline pain severity and pain interference followed a similar pattern. That is, more severe pain and pain interference at baseline were each associated with lower likelihood of consuming alcohol at baseline. More severe pain at baseline predicted a slightly increased rate of decline in the amount of alcohol consumed over the next 8 years among participants who drank. With respect to drinking frequency, having more numerous painful medical conditions, more severe pain, and more debilitating pain at baseline were each associated with a lowered likelihood of drinking alcohol more often than once a week. Among individuals who drank at least weekly, all of the baseline pain predictors were associated with lower frequency of drinking, and all of them contributed to a faster rate of decline over the 8-year interval in weekly frequency of alcohol consumption.	Amount of alcohol was defined as the number of drinks per day consumed by participants, on days they drank, during the last 3 months. Frequency of alcohol consumption was defined as the number of days per week that alcohol was consumed, during the last 3 months.	Participants were asked about the number of experienced painful medical conditions (chest, joint, headache, or back pain) since their last HRS assessment. Pain severity asked how severe pain was at the time of study interview (from no pain to severe pain). Pain interference was a dichotomous question asking participants about the difficulty to perform their usual activities due to pain.

#### Pain as a motivator of alcohol use

One study examined pain as a motivator of drinking among 55 US adults aged ≥50 years who had HIV and chronic pain.^48^ Ecological momentary assessment methods were used to obtain once-daily assessments of past 24-hour pain and substance use (alcohol, cannabis, or other drug use). Results indicated that reports of any pain in the last 24 hours did not predict same-day alcohol use (number of alcoholic beverages consumed). However, daily rating of worst pain in the last 24 hours was a significant predictor of alcohol consumption, such that for every one-unit increase in worst pain beyond an individual’s personal mean, alcohol use increased by a quarter drink.

#### Use of alcohol to cope with pain

Six studies examined alcohol use for pain coping. One qualitative study conducted among 20 older adults (age ≥60) in Nepal revealed that older adults often report drinking to reduce pain.^55^ Quantitative work has found similar patterns, with one study revealing that over 1 in 5 older adults use alcohol for pain coping.^56^ Specifically, this study included 719 adults aged 55-65 with a history of alcohol use (individuals who had never consumed alcohol in their lives were excluded) who were recruited from the community in a western part of the United States. Although there was a significant rise in the proportion of individuals who abstained from alcohol over time, a total of 20% and 22% of older adults reported using alcohol to manage pain at the 10-year and 20-year follow-ups, respectively. Similarly high rates of alcohol use for pain coping were observed among a sample of 1634 community-dwelling adults aged ≥65 in the US state of Florida.^57^ Indeed, 13% reported drinking alcohol for jaw joint pain, 24% for face pain, 16% for oral sores, 14% for burning mouth, and 22% for toothache.

However, two other studies revealed lower rates of drinking for pain coping.^49^^,^^58^ One of these was a study of 294 Swedish adults aged >75 who have chronic pain, which found that only 3% of people living at home and 2% of people living in special accommodation reported using alcohol for pain management.^49^ Moreover, 4% of those living alone and 2% of people living with someone reported using alcohol to manage pain. The other study evaluated the factor structure of the Pain Medication Questionnaire, which includes an item asking participants to rate how often the following statement applies to them on a scale ranging from 0 (never) to 4 (always): “At times, I drink alcohol to help my pain”.^58^ Among 150 older adults (mean age = 72.7) recruited from pain management clinics and receiving opioid medications, the mean score on this item was 0.13, with both the median and mode equal to 0, indicating that participants generally denied using alcohol for pain relief.

There is also some evidence that the use of alcohol for pain coping may vary as a function of sex, engagement in hazardous alcohol use, and pain severity. For example, the aforementioned study of 1634 community-dwelling adults aged ≥65 in the US state of Florida found that male older adults are significantly more likely to report self-treating with alcohol than females for jaw joint, face pain, painful oral sores, and toothache pain.^57^ In addition, in a study of 401 adults aged 55-65, 37.9% of men who reported at least one drinking problem (eg, being intoxicated or drunk, alcohol dependence, or withdrawal) also reported using alcohol to manage pain, compared to 15.1% of those who did not report drinking problems.^59^ Similarly, 37.9% of women who reported drinking problems reported using alcohol to manage pain, compared to 12.5% of those who did not. Among those with moderate to very severe pain, 56.5% of men and 58.6% of women who reported drinking problems reported using alcohol to manage pain. Among both men and women, more bodily pain and more drinking problems at baseline were independent predictors of the use of alcohol to manage pain.

#### Pain as a risk factor for developing alcohol-related disorders

Three studies examined pain as a risk factor for the onset, progression, and maintenance of alcohol use and related disorders. In a longitudinal study of 1291 US adults aged 55-65 who reported at least occasional alcohol use, baseline number of painful medical conditions alone had no effect on the 10-year pattern of decline in drinking problems.^60^ However, interpersonal spouse/partner resources moderated the effect of painful medical conditions on 10-year change in drinking frequency, such that, among individuals with more spouse/partner resources, having more painful medical conditions at baseline hastened decline in drinking frequency, whereas they had no effect on drinking frequency of individuals with fewer spouse/partner resources. Age also moderated the relationship between baseline painful medical conditions and 10-year change in frequency of drinking problems, with painful conditions hastening the decline in drinking problem frequency in older (ie, age 61-65 at baseline) participants, but having no effect in younger (age 55-60) participants.

In a second study of 5446 US adults aged 55-65, more numerous painful medical conditions had no effect on the 8-year rate of change in participants’ likelihood of consuming alcohol.^16^ Effects of baseline pain severity and pain interference followed a similar pattern. That is, more severe pain and pain interference at baseline were each associated a with lower likelihood of consuming alcohol at baseline. More severe pain at baseline predicted a slightly increased rate of decline in alcohol consumption over the next 8 years among participants who drank. With respect to drinking frequency, having more numerous painful medical conditions, more severe pain, and more debilitating pain at baseline were each associated with a lowered likelihood of drinking alcohol more often than once a week. Among individuals who drank at least weekly, all of the baseline pain predictors were associated with lower frequency of drinking, and all of them contributed to a faster rate of decline over the 8-year interval in weekly frequency of alcohol consumption.

Finally, in a third study of 3105 men aged 50-65 who participated in the Health and Retirement Study, a history of frequent pain significantly increased the likelihood that older men would report decreasing alcohol consumption (vs moderate or at-risk drinking) at follow-up.^61^

## Discussion

This scoping review identified 15 studies describing interrelationships between pain and alcohol use among older adults. In the sections that follow, we discuss: (1) the state of the extant literature on bidirectional relationships between pain and alcohol use in older adults; (2) potential third variables that may influence these relationships; (3) clinical implications; and (4) limitations of the current evidence and directions for future research.

### Bidirectional relationships between alcohol use and pain in older adults

Results of this review extend research on bidirectional associations between pain and alcohol use to older adults. Although some findings from this review of older adults align with the reciprocal model of pain and addiction,^19^ we also observed key differences, which suggest that the interplay between pain and alcohol may differ between older versus younger adults and underscores the need for targeted research in the older adult population.

#### Effects of alcohol use on pain

Consistent with evidence of acute analgesic effects among general adult populations,^21^ one study found that older adults often report that alcohol is helpful for pain management.^49^ However, a more recent study conducted among a small sample of older adults with HIV found that using alcohol to cope did not predict pain relief.^48^ Although one possible interpretation of this finding is that alcohol may not provide meaningful analgesic effects in this specific population, it is also important to note that alcohol may reduce pain acutely without improving overall coping or perceptions of “relief”. Notably, no studies experimentally tested the analgesic effects of alcohol in older adults, specifically, despite prior calls for research examining the effects of age on acute alcohol analgesia.^21^ Moreover, no studies examined the effects of acute alcohol abstinence on pain in older adults, despite reason to believe that pain reactivity may be exacerbated during alcohol withdrawal.^19^^,^^62^ Additional work is needed to systematically manipulate alcohol intoxication/deprivation to test the effects on pain reactivity in older adults.

In general adult samples, heavy alcohol use is associated with an increased risk for the onset, worsening, and maintenance of chronic pain,^31^^,^^63^^,^^64^ however, the current review identified mixed evidence in older adults. Although one study found that each additional alcohol drink per week increases an older person’s odds for poorer pain outcomes by ∼10%,^50^ another study found that alcohol use may instead be associated with a lower likelihood of chronic pain,^51^ and several studies found no association between alcohol use and chronic pain.^52-54^ Given that the relationship between alcohol consumption and the development of pain may be curvilinear,^20^ it is possible that the statistical models used in these studies did not fully capture the complexity of this relationship or identify levels of consumption at which the direction of effects changes. Indeed, there is reason to believe that hazardous alcohol consumption would increase the risk for the development and worsening of chronic pain in older adults. One prior study of older adults with HIV and chronic pain found that higher daily alcohol consumption predicted greater ratings of daily worst pain (ie, pain at its worst in the past 24 hours), suggesting that alcohol use may contribute to heightened pain severity.^48^ There is also evidence that heavy drinking can contribute to pathological changes to neural structures (eg, amygdala, prefrontal cortex, insula), perpetuated allostatic load in pain neurocircuitry, and central opioid deficiency.^19^^,^^20^ In addition, alcohol can reduce the effectiveness of pain treatments (eg, pharmacological/surgical interventions) through substance-specific (eg, cross-tolerance) and general neurobiological (eg, allostatic load) effects.^19^^,^^20^ Longitudinal studies that determine how various levels and patterns of alcohol consumption affect the development of chronic pain, utilization/effectiveness of pain treatments, and pain trajectories over time are needed.

#### Effects of pain on alcohol consumption

There is evidence that pain motivates alcohol use in older adults, with one study showing that for every one-unit increase in worst pain beyond an individual’s mean, alcohol use increases by a quarter drink.^48^ This finding is consistent with experimental findings that pain induction (vs a no-pain control condition) increases alcohol consumption.^24^ However, prior experimental work excluded participants aged 65 and older, thus, additional research is still needed to test pain as a causal motivator of alcohol consumption among older adults. In line with the hypothesis that pain can motivate alcohol use among older adults, the current review identified several studies suggesting that older adults often use alcohol for pain coping. Indeed, as many as 1 in 5 older adults use alcohol for pain coping.^56^ This finding is particularly alarming because individuals who rely more on alcohol to manage pain drink more frequently and in heavier amounts, report more warning signs of negative consequences of alcohol consumption, and have more drinking problems.^56^ Though one study found that older adults generally denied using alcohol for pain coping,^58^ it is important to note that this study comprised solely of patients receiving prescription opioids, who may be more likely to underreport alcohol use due to concerns about jeopardizing their access to pain medication.

Despite reason to believe that pain may contribute to the onset, progression, and maintenance of alcohol use/related disorders and interfere with alcohol cessation, only 3 studies examined this hypothesis. Moreover, each of these studies found that pain may actually be associated with a decline in drinking frequency among older adults.^16^^,^^60^^,^^61^ These findings differ from those observed in general adult samples, which have found that greater pain and pain-related functional impairment are associated with an increased likelihood of escalating from alcohol use to AUD.^65^ Moreover, evidence derived from general adult samples suggests that pain is associated with poorer rates of abstinence, drinking lapses, and greater post-intervention alcohol consumption.^66-68^ It is possible that older adults with pain are more likely to receive advice from their medical providers to reduce or quit drinking, and that this may explain the inverse association observed between pain and alcohol use in this population. It is also possible that high rates of comorbid medical conditions and concomitant medications in older adults^69^ may further influence the effects of pain on alcohol use in this population. Additional comparative studies are needed to examine differences in older versus younger cohorts with more attention to comorbid conditions.

### Third variables

Prior work has underscored the importance of considering third variables, including sociodemographic factors and comorbid psychopathology, when examining interrelations between pain and alcohol use.^19^^,^^20^ Two studies identified in this review examined gender differences in the use of alcohol for pain coping, with one study finding that older men are more likely than women to report using alcohol to cope with pain,^57^ and another finding no gender-specific effects in use of alcohol for pain coping.^59^ Another study examined the moderating effects of demographic characteristics (ie, age, gender, race, marital status, education, income) on relationships between pain and alcohol use in older adults.^16^ This study revealed that, although overall alcohol use declined among study participants from 1996 to 2004, older adults who were African American and/or reported lower income were less likely to follow this trend when experiencing greater pain severity or interference. Aside from these 3 exceptions, most studies did not examine the role of sociodemographic factors or comorbid psychopathology. Instead, many accounted for these factors by including them as covariates in statistical analyses.^50-54^^,^^59-61^ Although covariation can help to clarify unique variance in bidirectional pain-alcohol relations, it does little to clarify whether these variables function as underlying mechanisms driving these associations.

It is also likely that there are third variables underlying pain-alcohol comorbidity that are unique to older adults. For example, complex interactions among multimorbidity, pain, and alcohol use are particularly salient in later life, as 80% of those aged ≥65 have ≥2 chronic conditions.^33^ It is possible that older adults with multiple health conditions may reduce their drinking due to medical advice or the “sick quitter” effect. Alternatively, these same conditions may exacerbate pain and increase the perceived need to use alcohol to cope. Although several studies included in this review controlled for comorbid conditions or number of comorbidities,^50^^,^^51^^,^^54^^,^^56^ none explicitly examined these factors as mediators or moderators of pain–alcohol associations. Similarly, no studies explored the role of cognitive function, which is surprising given that cognitive decline could alter risk perception, coping strategies, and the capacity for self-regulation in both pain and alcohol use contexts.^70^

Loneliness and social isolation also warrant attention as potential third variables. One study included in this review found that living alone (vs living with someone) did not influence the use of alcohol for pain management,^49^ and another controlled for social isolation in examining risk factors for chronic pain.^51^ Additional work is clearly needed to better understand how social isolation influences associations between pain and alcohol use.

Additional work is also needed to explore the role of drinking history. Older adults often have complex drinking histories, including the potential for prior alcohol-related diagnoses, treatment histories, or long-term abstinence, all of which may shape current pain coping behaviors and the effects of alcohol use on pain outcomes. One study included in this review found that problem drinking was associated with a greater likelihood of using alcohol to manage pain,^59^ and another controlled for history of alcohol problems.^61^ Future research is needed to more comprehensively assess lifetime alcohol use patterns, including history of alcohol use, treatment, and periods of abstinence, to better understand how these trajectories relate to current experiences of pain and drinking behavior in older adults.

Finally, prior research has highlighted the importance of identifying transdiagnostic factors that may influence bidirectional pain-alcohol effects and represent promising targets for intervention development.^71^ Notably, several transdiagnostic constructs have emerged as promising candidates, including anxiety sensitivity, distress intolerance, pain-related anxiety, and pain catastrophizing. For instance, pain-related anxiety has been linked to greater alcohol-related consequences and an increased likelihood of alcohol-opioid co-use among adults with chronic low back pain.^72^ However, despite their theoretical and empirical relevance, none of the studies included in this review examined the potential role of these transdiagnostic factors in shaping bidirectional pain–alcohol associations.

### Clinical implications

Despite mixed findings and the need for additional research, results from this scoping review suggest that pain and alcohol use share a complex and nuanced relationship that should be considered when treating either condition. Clinicians working with older adults should assess both pain (eg, chronic pain status, pain intensity, physical impairment) and alcohol use when providing treatment for either condition. In the context of alcohol use, pain and related factors should be monitored throughout treatment. In the context of pain, it is important to consider patterns of alcohol use when evaluating and treating clinical pain. It will also be important for future research to prioritize the development and refinement of integrated intervention strategies that address co-occurring pain and alcohol use specifically in older adults. Although initial work has found that age-tailored substance use interventions may simultaneously lead to reductions in alcohol use and pain,^73^ additional work is still needed.

### Limitations and future research directions

An important limitation of this scoping review is the heterogeneity of the included studies in terms of age-related eligibility criteria. Because we did not apply strict age cutoffs, our sample encompassed studies with varying definitions of “older adults”, with the majority of studies (56%) including samples that were not limited to those aged 65 and older. Our decision to use a more inclusive search strategy was informed by evidence that aging-related vulnerabilities may be influenced by social, cultural, and economic factors and emerge earlier in populations with chronic conditions such as cancer or HIV, and by our desire to capture a broad range of evidence given the nascent state of research in this area. However, this approach introduced variability as there was substantial heterogeneity of the populations represented across studies. Indeed, younger cohorts of older adults (eg, those aged 50–64) may differ in important ways from those aged 65 and older, including potential differences in employment status, functional abilities, social roles, and health-related factors. These factors could meaningfully influence the relationship between chronic pain and alcohol use and thus warrant further exploration. Furthermore, although some included studies recruited participants for whom biological aging may occur earlier (eg, due to chronic illness), additional research is still needed to examine the differential effects of biological age (ie, a marker of aging that considers biological and physiological development factors, such as genetics, lifestyle, and comorbidities) and chronological age (ie, the amount of time a person has been alive) on bidirectional effects of pain and alcohol use. Additional work should also prioritize the recruitment of individuals who fall within the National Institutes of Health’s categorization of older adults (ie, aged ≥65).

Several additional limitations of this scoping review should be noted. First, there was significant heterogeneity in terms of how alcohol use (eg, current use, frequency, quantity, hazardous use) and pain (eg, pain ratings, chronic pain status, pain interference) were defined and assessed across studies, which poses challenges in comparing findings across studies and in drawing generalizable conclusions. Future research must prioritize the use of consistent, well-defined, validated measurement strategies. Additional research should also seek to understand how pain type, duration, and location may influence its relations with alcohol use. Second, generational differences can substantially influence attitudes, norms, and behaviors related to substance use.^74^ In addition to generational cohort effects, year of data collection may have also influenced study findings. For example, the Coronavirus disease 2019 (COVID-19) pandemic was associated with higher rates of anxiety/depression, social isolation, and loneliness,^75-77^ all of which are known risk factors for both chronic pain and alcohol use in older adults.^78^^,^^79^ Future research should explicitly account for generational cohort and period effects when examining pain and alcohol use in older adults. Third, we elected to include studies conducted across the world. However, there were not enough studies identified to examine cross-cultural differences. Given the potential influence of cross-cultural variance in drinking norms, motives, and behaviors^80^ and pain prevalence, beliefs, and coping responses,^81-83^ additional work is needed to determine whether differences in bidirectional pain–alcohol associations exist as a function of country/culture/context. Fourth, longitudinal studies examining interrelationships between pain and alcohol use may be affected by attrition due to mortality, and attrition may be even greater among older adults who have histories of heavier drinking, more severe pain, and multimorbidity. This has the potential to bias results and limit generalizability. Despite these limitations, the current scoping review represents an important contribution to the growing literature on bidirectional relationships between pain and alcohol use among older adults.

## Supplementary Material

glaf258_Supplementary_Data

## References

[1] Kuerbis A. Substance use among older adults: an update on prevalence, etiology, assessment, and intervention. Gerontology. 2020;66:249-258. 10.1159/000504363 31812954

[2] Kuerbis A , Sacco P, Blazer DG, Moore AA. Substance abuse among older adults. Clin Geriatr Med. 2014;30:629-654. 10.1016/j.cger.2014.04.008 25037298 PMC4146436

[3] Moore AA , Karno MP, Grella CE, et al. Alcohol, tobacco, and nonmedical drug use in older U.S. Adults: data from the 2001/02 national epidemiologic survey of alcohol and related conditions. J American Geriatrics Society. 2009;57:2275-2281. 10.1111/j.1532-5415.2009.02554.x PMC3646625 19874409

[4] LaRowe LR , Miaskowski C, Miller A, et al. Prevalence and sociodemographic correlates of chronic pain among a nationally representative sample of older adults in the United States. J Pain. 2024;25:104614. 10.1016/j.jpain.2024.104614 38936750 PMC11402580

[5] Domenichiello AF , Ramsden CE. The silent epidemic of chronic pain in older adults. Prog Neuropsychopharmacol Biol Psychiatry. 2019;93:284-290. 10.1016/j.pnpbp.2019.04.006 31004724 PMC6538291

[6] Wilkie R , Tajar A, McBeth J. The onset of widespread musculoskeletal pain is associated with a decrease in healthy ageing in older people: a population-based prospective study. PLoS One. 2013;8:e59858. 10.1371/journal.pone.0059858 23555810 PMC3612101

[7] Whitlock EL , Diaz-Ramirez LG, Glymour MM, Boscardin WJ, Covinsky KE, Smith AK. Association between persistent pain and memory decline and dementia in a longitudinal cohort of elders. JAMA Intern Med. 2017;177:1146-1153. 10.1001/jamainternmed.2017.1622 28586818 PMC5588896

[8] Saunders JB , Aasland OG, Babor TF, de la Fuente JR, Grant M. II. Development of the Alcohol Use Disorders Identification Test (AUDIT): WHO collaborative project on early detection of persons with harmful alcohol consumption. Addiction. 1993;88:791-804. 10.1111/j.1360-0443.1993.tb02093.x 8329970

[9] Grant BF , Chou SP, Saha TD, et al. Prevalence of 12-month alcohol use, high-risk drinking, and DSM-IV alcohol use disorder in the United States, 2001-2002 to 2012-2013: results from the National Epidemiologic Survey on alcohol and related conditions. JAMA Psychiatry. 2017;74:911-923. 10.1001/jamapsychiatry.2017.2161 28793133 PMC5710229

[10] Rehm J , Hasan OSM, Black SE, Shield KD, Schwarzinger M. Alcohol use and dementia: a systematic scoping review. Alzheimers Res Ther. 2019;11:1. 10.1186/s13195-018-0453-0 30611304 PMC6320619

[11] Moore AA , Endo JO, Carter MK. Is there a relationship between excessive drinking and functional impairment in older persons? J Am Geriatr Soc. 2003;51:44-49. 10.1034/j.1601-5215.2002.51008.x 12534844

[12] Blazer DG , Wu LT. The epidemiology of alcohol use disorders and subthreshold dependence in a middle-aged and elderly community sample. Am J Geriatr Psychiatry. 2011;19:685-694. 10.1097/JGP.0b013e3182006a96 21785289 PMC3144522

[13] LaRowe LR , Granados HC, Philpotts LL, Vranceanu AM, Ritchie CS. Prevalence of alcohol use among U.S. older adults with pain: a scoping review. Ageing Res Rev. 2024;101:102541. 10.1016/j.arr.2024.102541 39395578

[14] LaRowe LR , Miller A, Shah SJ, Ritchie CS. Hazardous alcohol use among community-dwelling older adults with persistent or recurrent pain: findings from the Health and Retirement Study. J Gerontol A Biol Sci Med Sci. 2024;79:glad281. 10.1093/gerona/glad281 38135282 PMC10959438

[15] Assari S , Smith JL, Saqib M, Bazargan M. Binge drinking among economically disadvantaged African American older adults with diabetes. Behav Sci (Basel). 2019;9:97. 10.3390/bs9090097 31514373 PMC6769764

[16] Brennan PL , Soohoo S. Pain and use of alcohol in later life: prospective evidence from the Health and Retirement Study. J Aging Health. 2013;25:656-677. 10.1177/0898264313484058 23640817 PMC3883439

[17] Hogans BB , Siaton BC, Taylor MN, Katzel LI, Sorkin JD. Low back pain and substance use: diagnostic and administrative coding for opioid use and dependence increased in U.S. older adults with low back pain. Pain Med. 2021;22:836-847. 10.1093/pm/pnaa428 33594426 PMC8599750

[18] Lasebikan VO , Gureje O. Lifetime and 7-day alcohol consumption in the elderly, prevalence and correlates: reports from the Ibadan Study of Aging. Afr J Med Med Sci. 2015;44:33-41. 26548114

[19] Ditre JW , Zale EL, LaRowe LR. A reciprocal model of pain and substance use: transdiagnostic considerations, clinical implications, and future directions. Annu Rev Clin Psychol. 2019;15:503-528. 10.1146/annurev-clinpsy-050718-095440 30566371

[20] Zale EL , Maisto SA, Ditre JW. Interrelations between pain and alcohol: an integrative review. Clin Psychol Rev. 2015;37:57-71. 10.1016/j.cpr.2015.02.005 25766100 PMC4385458

[21] Thompson T , Oram C, Correll CU, Tsermentseli S, Stubbs B. Analgesic effects of alcohol: a systematic review and meta-analysis of controlled experimental studies in healthy participants. J Pain. 2017;18:499-510. 10.1016/j.jpain.2016.11.009 27919773

[22] Holmes A , Williamson O, Hogg M, et al. Predictors of pain severity 3 months after serious injury. Pain Med. 2010;11:990-1000. 10.1111/j.1526-4637.2010.00890.x 20642728

[23] Castillo RC , MacKenzie EJ, Wegener ST, Bosse MJ, LEAP Study Group. Prevalence of chronic pain seven years following limb threatening lower extremity trauma. Pain. 2006;124:321-329. 10.1016/j.pain.2006.04.020 16781066

[24] Ditre JW , LaRowe LR, Powers JM, et al. Pain as a causal motivator of alcohol consumption: associations with gender and race. J Psychopathol Clin Sci. 2023;132:101-109. 10.1037/abn0000792 36480413 PMC9870930

[25] Moskal D , Maisto SA, De Vita M, Ditre JW. Effects of experimental pain induction on alcohol urge, intention to consume alcohol, and alcohol demand. Exp Clin Psychopharmacol. 2018;26:65-76. 10.1037/pha0000170 29323505 PMC5794517

[26] Riley JL 3rd , King C. Self-report of alcohol use for pain in a multi-ethnic community sample. J Pain. 2009;10:944-952. 10.1016/j.jpain.2009.03.005 19712901 PMC2734914

[27] Karimi R , Mallah N, Nedjat S, Beasley MJ, Takkouche B. Association between alcohol consumption and chronic pain: a systematic review and meta-analysis. Br J Anaesth. 2022;129:355-365. 10.1016/j.bja.2022.03.010 35410791

[28] Edwards S , Vendruscolo LF, Gilpin NW, Wojnar M, Witkiewitz K. Alcohol and pain: a translational review of preclinical and clinical findings to inform future treatment strategies. Alcohol Clin Exp Res. 2020;44:368-383. 10.1111/acer.14260 31840821 PMC11004915

[29] Fitzpatrick-Schmidt T , Edwards S. Within and beyond the binary: sex and gender differences in pain and alcohol use disorder. Curr Addict Rep. 2024;11:68-80. 10.1007/s40429-023-00534-y 40747361 PMC12312634

[30] Horn-Hofmann C , Büscher P, Lautenbacher S, Wolstein J. The effect of nonrecurring alcohol administration on pain perception in humans: a systematic review. J Pain Res. 2015;8:175-187. Published 2015 Apr 23. 10.2147/JPR.S79618 25960674 PMC4412487

[31] Ferreira PH , Pinheiro MB, Machado GC, Ferreira ML. Is alcohol intake associated with low back pain? A systematic review of observational studies. Man Ther. 2013;18:183-190. 10.1016/j.math.2012.10.007 23146385

[32] LaRowe LR , Pham T, Szapary C, Vranceanu AM. Shaping the future of geriatric chronic pain care: a research agenda for progress. Pain Manag. 2025;15:265-277. 10.1080/17581869.2025.2493609 40246703 PMC12118399

[33] National Council on Aging. Chronic inequities: measuring disease cost burden among older adults in the U.S.A Health and Retirement Study Analysis. 2022. Accessed January 9, 2024. Available from: https://ncoa.org/article/the-inequities-in-the-cost-of-chronic-disease-why-it-matters-for-older-adults.

[34] Charlesworth CJ , Smit E, Lee DS, Alramadhan F, Odden MC. Polypharmacy among adults aged 65 years and older in the United States: 1988-2010. J Gerontol A Biol Sci Med Sci. 2015;70:989-995. 10.1093/gerona/glv013 25733718 PMC4573668

[35] Trifirò G , Spina E. Age-related changes in pharmacodynamics: focus on drugs acting on central nervous and cardiovascular systems. Curr Drug Metab. 2011;12:611-620. 10.2174/138920011796504473 21495972

[36] Mangoni AA , Jackson SH. Age-related changes in pharmacokinetics and pharmacodynamics: basic principles and practical applications. Br J Clin Pharmacol. 2004;57:6-14. 10.1046/j.1365-2125.2003.02007.x 14678335 PMC1884408

[37] Tupler LA , Hege S, Ellinwood EH. Jr. Alcohol pharmacodynamics in young-elderly adults contrasted with young and middle-aged subjects. Psychopharmacology (Berl). 1995;118:460-470. 10.1007/BF02245947 7568633

[38] Sklar AL , Gilbertson R, Boissoneault J, Prather R, Nixon SJ. Differential effects of moderate alcohol consumption on performance among older and younger adults. Alcohol Clin Exp Res. 2012;36:2150-2156. 10.1111/j.1530-0277.2012.01833.x 22591190 PMC3424337

[39] Yarnell S , Li L, MacGrory B, Trevisan L, Kirwin P. Substance use disorders in later life: a review and synthesis of the literature of an emerging public health concern. Am J Geriatr Psychiatry. 2020;28:226-236. 10.1016/j.jagp.2019.06.005 31340887

[40] Gilbertson R , Ceballos NA, Prather R, Nixon SJ. Effects of acute alcohol consumption in older and younger adults: perceived impairment versus psychomotor performance. J Stud Alcohol Drugs. 2009;70:242-252. 10.15288/jsad.2009.70.242 19261236 PMC2653610

[41] Ahangari A , Stewart Williams J, Myléus A. Pain and alcohol consumption among older adults: findings from the World Health Organization Study on global AGEing and adult health, Wave 1. Trop Med Int Health. 2016;21:1282-1292. 10.1111/tmi.12757 27443945

[42] Fink A , Morton SC, Beck JC, et al. The alcohol-related problems survey: identifying hazardous and harmful drinking in older primary care patients. J Am Geriatr Soc. 2002;50:1717-1722. 10.1046/j.1532-5415.2002.50467.x 12366628

[43] Peters MD. The Joanna Briggs Institute Reviewers’ Manual 2015: methodology for JBI scoping reviews. 2015. Available from: https://reben.com.br/revista/wp-content/uploads/2020/10/Scoping.pdf

[44] Tricco AC , Lillie E, Zarin W, et al. PRISMA Extension for Scoping Reviews (PRISMA-ScR): checklist and explanation. Ann Intern Med. 2018;169:467-473. 10.7326/M18-0850 30178033

[45] LaRowe LR. Interrelations between pain and alcohol use among older adults: a scoping review. 2024. 10.17605/OSF.IO/KNVCE PMC12758971 41269122

[46] *Covidence Systematic Review Software*. Available from: www.covidence.org

[47] Peltzer K. Lifestyle factors, mental health, and incident and persistent intrusive pain among ageing adults in South Africa. Scand J Pain. 2023;23:161-167. 10.1515/sjpain-2022-0013 35467093 PMC10249480

[48] Kuerbis A , Reid MC, Lake JE, et al. Daily factors driving daily substance use and chronic pain among older adults with HIV: an exploratory study using ecological momentary assessment. Alcohol. 2019;77:31-39. 10.1016/j.alcohol.2018.10.003 30308287 PMC6456439

[49] Jakobsson U , Rahm Hallberg I, Westergren A. Pain management in elderly persons who require assistance with activities of daily living: a comparison of those living at home with those in special accommodations. Eur J Pain. 2004;8:335-344. 10.1016/j.ejpain.2003.10.007 15207514

[50] Parreira PCS , Maher CG, Ferreira ML, et al. A longitudinal study of the influence of comorbidities and lifestyle factors on low back pain in older men. Pain. 2017;158:1571-1576. 10.1097/j.pain.0000000000000952 28520648

[51] Fancourt D , Steptoe A. Physical and psychosocial factors in the prevention of chronic pain in older age. J Pain. 2018;19:1385-1391. 10.1016/j.jpain.2018.06.001 29949780 PMC6288062

[52] Caplan L , Pittman CB, Zeringue AL, et al. An observational study of musculoskeletal pain among patients receiving bisphosphonate therapy. Mayo Clin Proc. 2010;85:341-348. 10.4065/mcp.2009.0492 20231335 PMC2848422

[53] Marttinen MK , Kautiainen H, Vuorimaa H, Kauppi MJ. Pain experience in an aging adult population during a 10-year follow-up. Scand J Pain. 2021;21:716-723. 10.1515/sjpain-2021-0061 34114388

[54] Li R , Dworkin RH, Chapman BP, et al. Moderate to severe chronic pain in later life: risk and resilience factors for recovery. J Pain. 2021;22:1657-1671. 10.1016/j.jpain.2021.05.007 34174387

[55] Begam S , Paudel S, Chalise A, Khan GM, Tuladhar L, Khadka S. Exploring the sociodemographic factors and consequences related to alcohol consumption among older indigenous community of a district in Nepal: a qualitative study. J Nepal Med Assoc. 2024;62:188-195. 10.31729/jnma.8495 PMC10924484 39356786

[56] Moos RH , Brennan PL, Schutte KK, Moos BS. Older adults’ health and late-life drinking patterns: a 20-year perspective. Aging Ment Health. 2010;14:33-43. 10.1080/13607860902918264 20155519

[57] Riley JL 3rd , Gilbert GH, Heft MW. Orofacial pain: racial and sex differences among older adults. J Public Health Dent. 2002;62:132-139. 10.1111/j.1752-7325.2002.tb03434.x 12180040

[58] Park J , Clement R, Lavin R. Factor structure of pain medication questionnaire in community-dwelling older adults with chronic pain. Pain Pract. 2011;11:314-324. 10.1111/j.1533-2500.2010.00422.x 21143370

[59] Brennan PL , Schutte KK, Moos RH. Pain and use of alcohol to manage pain: prevalence and 3-year outcomes among older problem and non-problem drinkers. Addiction. 2005;100:777-786. 10.1111/j.1360-0443.2005.01074.x 15918808

[60] Brennan PL , Schutte KK, SooHoo S, Moos RH. Painful medical conditions and alcohol use: a prospective study among older adults. Pain Med. 2011;12:1049-1059. 10.1111/j.1526-4637.2011.01156.x 21668742 PMC3146463

[61] Bobo JK , Greek AA, Klepinger DH, Herting JR. Predicting 10-year alcohol use trajectories among men age 50 years and older. Am J Geriatr Psychiatry. 2013;21:204-213. 10.1016/j.jagp.2012.10.021 23343494

[62] Jochum T , Boettger MK, Burkhardt C, Juckel G, Bär KJ. Increased pain sensitivity in alcohol withdrawal syndrome. Eur J Pain. 2010;14:713-718. 10.1016/j.ejpain.2009.11.008 20018536

[63] Cheng Y , Macera CA, Davis DR, Ainsworth BE, Troped PJ, Blair SN. Physical activity and self-reported, physician-diagnosed osteoarthritis: is physical activity a risk factor? J Clin Epidemiol. 2000;53:315-322. 10.1016/s0895-4356(99)00168-7 10760643

[64] Sá KN , Baptista AF, Matos MA, Lessa. Chronic pain and gender in Salvador population, Brazil. Pain. 2008;139:498-506. 10.1016/j.pain.2008.06.008 18672325

[65] McDermott KA , Joyner KJ, Hakes JK, Okey SA, Cougle JR. Pain interference and alcohol, nicotine, and cannabis use disorder in a national sample of substance users. Drug Alcohol Depend. 2018;186:53-59. 10.1016/j.drugalcdep.2018.01.011 29550622

[66] Caldeiro RM , Malte CA, Calsyn DA, et al. The association of persistent pain with out-patient addiction treatment outcomes and service utilization. Addiction. 2008;103:1996-2005. 10.1111/j.1360-0443.2008.02358.x 18855809

[67] Larson MJ , Paasche-Orlow M, Cheng DM, Lloyd-Travaglini C, Saitz R, Samet JH. Persistent pain is associated with substance use after detoxification: a prospective cohort analysis. Addiction. 2007;102:752-760. 10.1111/j.1360-0443.2007.01759.x 17506152

[68] Witkiewitz K , Vowles KE, McCallion E, Frohe T, Kirouac M, Maisto SA. Pain as a predictor of heavy drinking and any drinking lapses in the COMBINE study and the UK Alcohol Treatment Trial. Addiction. 2015;110:1262-1271. 10.1111/add.12964 25919978 PMC4503502

[69] Schneider J , Algharably EAE, Budnick A, Wenzel A, Dräger D, Kreutz R. High Prevalence of multimorbidity and polypharmacy in elderly patients with chronic pain receiving home care are associated with multiple medication-related problems. Front Pharmacol. 2021;12:686990. 10.3389/fphar.2021.686990 34168565 PMC8217758

[70] Zhao L , Zhang L, Tang Y, Tu Y. Cognitive impairments in chronic pain: a brain aging framework. Trends Cogn Sci. 2025;29:570-585. 10.1016/j.tics.2024.12.004 39753445

[71] Krueger RF , Eaton NR. Transdiagnostic factors of mental disorders. World Psychiatry. 2015;14:27-29. 10.1002/wps.20175 25655146 PMC4329885

[72] LaRowe LR , Powers JM, Garey L, Rogers AH, Zvolensky MJ, Ditre JW. Pain-related anxiety, sex, and co-use of alcohol and prescription opioids among adults with chronic low back pain. Drug Alcohol Depend. 2020;214:108171. 10.1016/j.drugalcdep.2020.108171 32679522 PMC7423687

[73] Hafford-Letchfield T , McQuarrie T, Clancy C, Thom B, Jain B. Community based interventions for problematic substance use in later life: a systematic review of evaluated studies and their outcomes. Int J Environ Res Public Health. 2020;17:7994. 10.3390/ijerph17217994 33143159 PMC7663344

[74] Ashford RD , Brown A. Bridging the gaps: intergenerational findings from the substance use disorder and recovery field. J Intergener Relat. 2017;15:326-351. 10.1080/15350770.2017.1368326

[75] Wu B. Social isolation and loneliness among older adults in the context of COVID-19: a global challenge. Glob Health Res Policy. 2020;5:27. 10.1186/s41256-020-00154-3 32514427 PMC7272234

[76] Lebrasseur A , Fortin-Bédard N, Lettre J, et al. Impact of the COVID-19 pandemic on older adults: rapid review. JMIR Aging. 2021;4:e26474. 10.2196/26474 33720839 PMC8043147

[77] Gosselin P , Castonguay C, Goyette M, et al. Anxiety among older adults during the COVID-19 pandemic. J Anxiety Disord. 2022;92:102633. 10.1016/j.janxdis.2022.102633 36115079 PMC9465474

[78] Miaskowski C , Blyth F, Nicosia F, et al. A biopsychosocial model of chronic pain for older adults. Pain Med. 2020;21:1793-1805. 10.1093/pm/pnz329 31846035

[79] Kelly S , Olanrewaju O, Cowan A, Brayne C, Lafortune L. Alcohol and older people: a systematic review of barriers, facilitators and context of drinking in older people and implications for intervention design. PLoS One. 2018;13:e0191189. 10.1371/journal.pone.0191189 29370214 PMC5784942

[80] Mackinnon SP , Couture ME, Cooper ML, et al.; DRINC Team. Cross-cultural comparisons of drinking motives in 10 countries: data from the DRINC project. Drug Alcohol Rev. 2017;36:721-730. 10.1111/dar.12464 28337801

[81] Ferreira-Valente MA , Pais-Ribeiro JL, Jensen MP. Associations between psychosocial factors and pain intensity, physical functioning, and psychological functioning in patients with chronic pain: a cross-cultural comparison. Clin J Pain. 2014;30:713-723. 10.1097/AJP.0000000000000027 24042349

[82] Moore R , Brødsgaard I, Cross-cultural investigations of pain. In: IK Crombie, PR Croft, SJ Linton, eds. Epidemiology of Pain. 1999:53-80.

[83] Rajkumar RP. The influence of cultural and religious factors on cross-national variations in the prevalence of chronic back and neck pain: an analysis of data from the global burden of disease 2019 study. Front Pain Res (Lausanne). 2023;4:1189432. 10.3389/fpain.2023.1189432 37305205 PMC10248050

