# Supplementary material for: Bidirectional relationships between pain and alcohol use among older adults: a scoping review
Source: J Gerontol A Biol Sci Med Sci. 2025 Nov 21;81(1):glaf258. doi: 10.1093/gerona/glaf258 (PMC12758971; doi:10.1093/gerona/glaf258)
Supplement: glaf258_Supplementary_Data [file glaf258_supplementary_data.docx]

**Appendix 1**

**Details Regarding Search Strategy**

**Database:**
Ovid MEDLINE(R) Epub Ahead of Print, In-Process & Other Non-Indexed Citations, Ovid MEDLINE(R) Daily, Ovid MEDLINE and Versions(R) 1946 to Present

| **#** | **Query** |
| --- | --- |
| 1 | exp Aged/ or exp geriatrics/ or health services for the aged/ |
| 2 | (aged or geriatric* or elder* or older or ageing or aging).ti,ab. |
| 3 | 1 or 2 |
| 4 | (pain or painful).ti,ab. |
| 5 | exp pain/ or pain management/ or exp pain perception/ or pain clinics/ or pain measurement/ |
| 6 | 4 or 5 |
| 7 | alcohol*.ti,ab. |
| 8 | exp alcohol related disorders/ or exp drinking behavior/ or exp alcoholic beverages/ |
| 9 | 7 or 8 |
| 10 | 3 and 6 and 9 |
| 11 | exp animals/ not humans/ |
| 12 | 10 not 11 |
| 13 | limit 12 to english language |
| 14 | limit 13 to (comment or editorial or letter or overall) |
| 15 | 13 not 14 |

**Database:**
Cochrane Central Register of Controlled Trials

| **#** | **Query** |
| --- | --- |
| 1 | exp Aged/ or exp geriatrics/ or health services for the aged/ |
| 2 | (aged or geriatric* or elder* or older or ageing or aging).ti,ab. |
| 3 | 1 or 2 |
| 4 | (pain or painful).ti,ab. |
| 5 | exp pain/ or pain management/ or exp pain perception/ or pain clinics/ or pain measurement/ |
| 6 | 4 or 5 |
| 7 | alcohol*.ti,ab. |
| 8 | exp alcohol related disorders/ or exp drinking behavior/ or exp alcoholic beverages/ |
| 9 | 7 or 8 |
| 10 | 3 and 6 and 9 |
| 11 | exp animals/ not humans/ |
| 12 | 10 not 11 |
| 13 | limit 12 to english language |

**Database:**
PsycInfo via Ovid

| **#** | **Query** |
| --- | --- |
| 1 | (aged or geriatric* or elder* or older or ageing or aging).ti,ab. |
| 2 | (pain or painful).ti,ab. |
| 3 | alcohol*.ti,ab. |
| 4 | Pain Management/ or exp Pain Perception/ or Pain Measurement/ or exp Pain/ |
| 5 | exp alcohol drinking patterns/ |
| 6 | exp "alcohol use disorder"/ |
| 7 | exp Alcoholic Beverages/ |
| 8 | 2 or 4 |
| 9 | 3 or 5 or 6 or 7 |
| 10 | 1 and 8 and 9 |
| 11 | 8 and 9 |
| 12 | limit 11 to ("380 aged " or "390 very old ") |
| 13 | 10 or 12 |
| 14 | limit 13 to english language |
| 15 | limit 14 to animal |
| 16 | 14 not 15 |
| 17 | limit 16 to (chapter or "column/opinion" or "comment/reply" or editorial or letter) |
| 18 | 16 not 17 |

**Database:**
CINAHL via Ebsco

| **#** | **Query** |
| --- | --- |
| S1 | TI ( (aged or geriatric* or elder* or older or ageing or aging) ) OR AB ( (aged or geriatric* or elder* or older or ageing or aging) ) |
| S2 | (MH "Aged+") OR (MH "Health Services for Older Persons") OR (MH "Gerontologic Nursing+") OR (MH "Gerontologic Care") |
| S3 | S1 OR S2 |
| S4 | TI ( pain or painful ) OR AB ( pain or painful ) |
| S5 | (MH "Pain+") OR (MH "Pain Clinics") OR (MH "Pain Measurement") OR (MH "Pain Management") |
| S6 | S4 OR S5 |
| S7 | TI alcohol* OR AB alcohol* |
| S8 | (MH "Drinking Behavior+") OR (MH "Alcohol-Related Disorders+") OR (MH "Alcoholic Beverages+") |
| S9 | S7 OR S8 |
| S10 | S3 AND S6 AND S9 |
| S11 | S3 AND S6 AND S9 Limiters - English Language |
| S12 | (MH "Animals+") NOT (MH human) |
| S13 | S11 NOT S12 |
| S14 | S11 NOT S12 Publication Type: Book, Book Chapter, Book Review, Commentary, Editorial, Letter, Proceedings |
| S15 | S13 NOT S14 |

**Database:**
Embase

| # | Query |
| --- | --- |
| #1 | aged:ab,ti OR geriatric*:ab,ti OR elder*:ab,ti OR older:ab,ti OR ageing:ab,ti OR aging:ab,ti |
| #2 | 'aged'/exp |
| #3 | #1 OR #2 |
| #4 | 'pain'/mj/exp OR 'pain measurement'/de |
| #5 | pain:ab,ti OR painful:ab,ti |
| #6 | #4 OR #5 |
| #7 | alcohol*:ab,ti |
| #8 | 'alcoholism'/exp OR 'alcoholic beverage'/exp OR 'alcohol consumption'/exp |
| #9 | #7 OR #8 |
| #10 | #3 AND #6 AND #9 |
| #11 | #3 AND #6 AND #9 AND [humans]/lim |
| #12 | #3 AND #6 AND #9 AND [humans]/lim AND [english]/lim |
| #13 | #12 AND ('Conference Abstract'/it OR 'Conference Paper'/it OR 'Conference Review'/it OR 'Letter'/it OR 'Note'/it) |
| #14 | #12 NOT #13 |

**Web of Science Core Collection**

| **#** | **Query** |
| --- | --- |
| 1 | (aged or geriatric* or elder* or older or ageing or aging) (Topic) |
| 2 | (pain or painful) (Topic) |
| 3 | alcohol* (Topic) |
| 4 | #3 AND #2 AND #1 |
| 5 | #3 AND #2 AND #1 and English (Exclude – Languages) and Proceeding Paper or Editorial Material or Book Chapters or Note or Meeting Abstract or Letter or Meeting (Exclude – Document Types) |
| 6 | #3 AND #2 AND #1 and Proceeding Paper or Editorial Material or Book Chapters or Note or Meeting Abstract or Letter or Meeting (Exclude – Document Types) |
| 7 | #3 AND #2 AND #1 and Proceeding Paper or Editorial Material or Book Chapters or Note or Meeting Abstract or Letter or Meeting (Exclude – Document Types) and English (Languages) |
| 8 | animal OR animals OR mouse OR mice OR rat OR rats (Topic) |
| 9 | #7 NOT #8 |
